# Combined Intranasal Insulin/Saxagliptin/Metformin Therapies Ameliorate the Effect of Combined Oral Contraceptive- (COC-) Induced Metabolic Syndrome (MetS) with a Major Target on Glucose Metabolism in Adult Female Wistar Rats

**DOI:** 10.1155/2021/9693171

**Published:** 2021-12-13

**Authors:** Saheed Olanrewaju Afolabi, Joy Folahan, Olalekan Agede, Olufunke Olorundare

**Affiliations:** Department of Pharmacology and Therapeutics, University of Ilorin, Ilorin, Nigeria

## Abstract

**Objective:**

To evaluate the effect of the chronic use of combined oral contraceptives (COCs: ethinyl estradiol and levonorgestrel) on the indices of metabolic syndrome in adult female Wistar rats and possible therapeutic management.

**Materials and Methods:**

64 female Wistar rats received either distilled water, norethindrone (NOR), COC, intranasal insulin (INI), metformin (MET), saxagliptin (SAX), INI+MET, and INI+SAX. After 8 weeks of exposure to COC, the animals were sorted into the therapeutic groups. Several parameters were assayed for, such as body weight changes, fasting blood glucose (FBG) level, insulin levels, inflammatory cytokines, and glycated hemoglobin (Hb1Ac).

**Results:**

The levels of FBG, insulin, and Hb1Ac were increased consequent upon COC treatment. Treatment with INI+SAX and INI+MET reduced significantly the levels of FBG and Hb1Ac; in addition, the level of insulin was significantly increased in the INI+MET groups (*p* ≤ 0.05). Serum lipid profile analysis showed a statistical reduction in high-density lipoprotein (HDL) level; this reduction was also significantly reversed in the INI+SAX group. Reduced catalase activity observed in the COC group was reversed in the INI+MET group (*p* ≤ 0.05). A nonsignificant increase in the level of TNF-*α* as a result of COC treatment was reversed by INI and INI+MET treatment. Liver GLUT4 and G-6-phosphate levels were significantly increased by COC treatment, and this effect was reversed by INI+SAX in both assays, respectively (*p* ≤ 0.01).

**Conclusions:**

The use of MET and SAX in combination with INI has been shown to reverse some indices of MetS. This study proposes a clinical phase to backup and ascertain these preclinical findings.

## 1. Introduction

Premenopausal women are active users of hormonal contraceptives. In the last decade, there has been an upsurge in this usage; scientific reports have estimated that over 150 million sexually active women subscribe to these pharmaceuticals globally [1–3]. Commercially available formulations consisting of a synthetic estrogen and progestin constitute the globally recognized combined oral contraceptives (COCs) [4, 5]. In addition to its contraceptive effects, constant administration of COC is also being explored as a therapeutic intervention against polycystic ovary syndrome (PCOS) and dysmenorrhea [6, 7]. However, the use of COC has been linked with disruption in body weight regulation, arterial blood pressure, glucose metabolism, and general homeostatic regulations in females [8, 9]. Endocrinologists and researchers in the field of reproductive health suggest that continuous prescription of COC should be revised with respect to these adverse effects [10, 11]. Ethinyl estradiol (EE), synthesized from 17*β*-estradiol (E2), is the most used synthetic estrogen in COC [7, 12, 13]; it is an estrogen receptor agonist. Over the years, ethinyl estradiol has been used for menopausal symptoms, gynecological disorders, and some forms of hormone-sensitive cancers [14–16]. Currently, EE is almost exclusively employed for the adjunctive role it plays in the COC, making it the most widely used estrogen [17]. Levonorgestrel (LEV) is a use-alone emergency contraceptive pill and is also formulated with estrogen in some COC [18]. It is a progestin that works principally by preventing ovulation and enhancing the closure of the cervix to prevent the entry of sperm, similar to the hormone progesterone [19, 20]. LEV is a synthetic steroid (17*α*-ethynyl-18-methyl-19-nortestosterone) derived from testosterone [21].

The combination of these hormones has been studied for their therapeutic efficacy in women who have PCOS, a pathological condition characterized by dysregulation in and carbohydrate metabolism in women [22, 23]. Generally, COCs from its inception have been linked with dysregulation of glucose homeostasis. The COCs containing norethindrone and EE have reportedly increased glycemia levels in premenopausal women [24]; this effect was in tandem with COC containing levonorgestrel and EE [25].

Glycemic regulation is under the control of the hormone, insulin. When glucose level in the extracellular fluid increases, it is transported to the pancreatic *β*-cells and its consequent metabolism raises the intracellular ATP/ADP ratio. This closes the ATP-sensitive/K+ channels (KATP), causing depolarization of the *β*-cell membranes and activation of Ca^2+^-activated voltage channels; the Ca^2+^ influx then activates the release of insulin granules via exocytosis [26, 27]. Insulin resistance (IR) is a metabolic disorder that features a decreased response to both exogenous and endogenous insulin in cells, specific adipose tissues, skeletal muscles, and the whole body. IR is one of the major indicators and pathological driver of metabolic syndrome, a disease condition caused by a myriad of metabolic mishaps which features IR, hyperinsulinemia, glucose intolerance, and dyslipidemia [28, 29]. Metabolic syndrome is suggested to be the most important medical problem of the 21st century. Insulin resistance is a known risk factor for type 2 diabetes [30, 31]. Metformin is an oral hypoglycemic agent belonging to the biguanide class of antidiabetic agents. It is often used as first-line treatment of diabetes [32, 33]. It improves hyperglycemia primarily through its suppression of hepatic glucose production, increases insulin sensitivity, enhances peripheral glucose uptake, upregulates oxidation of fatty acid [34], and downregulates glucose absorption from the gastrointestinal tract. A member of the dipeptidyl peptidase-4 (DPP-4) inhibitor group of oral hypoglycemics, saxagliptin is approved by the US Food and Drug Administration and the European Medicines Agency. DPP-4 inhibitors are believed to be therapeutically effective in the management of IR and thus could prevent metabolic syndrome [35].

Here, we set out to investigate the effect of combined oral contraceptives (ethinyl estradiol and levonorgestrel) and minipill (norethindrone) on adult female albino rats; and the possible ameliorative effect of single/combined administration of saxagliptin, intranasal insulin and metformin on the metabolic syndrome status.

## 2. Materials and Methods

### 2.1. Experimental Groups

Adult female Wistar rats (64) were obtained from an animal house facility in Ogbomosho, Oyo state, Nigeria, and housed in groups of eight and allowed to acclimatize for 7 days in the animal housing facility of the Faculty of Basic Medical Sciences, University of Ilorin, Nigeria. Housing conditions were temperature of 25°C and a 12 : 12 light : dark cycle with light on by 07 : 00 and off at 1900. The animals were housed in metabolic cages with wood shavings as beddings replaced fortnightly. Animals had unrestricted access to rat pelletized feed from Top Feed Nigeria Ltd. (consisting of maize, soybean, GNC, corn brown, wheat offal, palm kernel cake, and bone) and water ad libitum. Animals were monitored and cleaned up daily. All eight groups received distilled water/NOR/COC for 8 weeks; then, each group received various therapeutic regimen for 7 days as outlined below. All protocols involving the use of experimental animals were duly approved by the University Ethical Committee with the approval number UERC/ASN/2020/2028. The international guide to handle and use of experimental animals by the National Research Council [36] and Committee for the Update of the Guide for the Care and Use of Laboratory Animals [37] was strictly adhered to during the animal handling phase of this study.

The animal experimental groupings are underlisted.

*Group 1*. Control

*Group 2*. Norethindrone (NOR), 1.4 mg/kg/day

*Group 3*. Combined oral contraceptives (EE and LEV; COC), 7.6 mg/kg/day

*Group 4*. COC+intranasal insulin (INI: 2 IU, 1 IU/10 *μ*l and 10 *μ*l/nostril)

*Group 5*. COC+metformin (MET: 150 mg/kg/day)

*Group 6*. COC+saxagliptin (SAX: 10 mg/kg/day)

*Group 7*. COC+INI+MET

*Group 8*. COC+INI+SAX

### 2.2. Drugs

Recombinant human insulin (actrapid, 100 IU/ml) was purchased from Novo Nordisk, combined oral contraceptives (ethinyl estradiol and levonorgestrel) were from Bayer USA, and metformin, norethindrone (minipill), and saxagliptin were purchased from MedPlus pharmacy Ilorin. All biochemical reagents including ELISA kits for rat insulin, glucose-6-phosphate, lipids, and glucokinase were purchased from Elabscience (US). All other chemicals were of analytical grade and commercially available. Accu-Chek glucometer and glucose strips were purchased from Roche Diagnostic, USA (Micropipette P-10, Eppendorf).

### 2.3. Intranasal Insulin Administration

According to the method described by Njan et al. [38], rats were anesthetized by placing them in tightly sealed transparent glass jars containing isoflurane (5%) obtained from Southern Anesthesia & Surgical, (West Columbia, US) for brief periods (<30 s), removed, and restrained in the supine position. A total of 2 IU (1 IU/10 *μ*l and 10 *μ*l/nostril) of rapid acting insulin or vehicle (0.9% Sodium Chloride, NaCl) was rapidly dispensed intranasally using a micropipette (P-10, Eppendorf). This procedure lasted for about 15 s. Animals became conscious in another 15–20 s and were observed physically before returning to the metabolic cages.

### 2.4. Measurement of Body Weight, Organosomatic Index, Fasting Blood Glucose, and Glucose Tolerance Test

Body weights of control and experimental rats were analyzed weekly throughout the experimental phase. At the end of the experimental period, the animals were sacrificed, the pancreas was harvested, and the organosomatic index was calculated (i.e., weight of the pancreas as a percentage of the animal body weight). Fasting blood glucose levels were analyzed using a glucometer (Accu-Chek Performa, Roche Diagnostic, USA). An oral glucose tolerance test (OGTT) was performed using an oral dose of glucose (2 g/kg) for 2 h after 8 consecutive weeks of treatment. Prior to the OGTT procedure, animals were fasted overnight and given only water to drink throughout the time. The blood samples were collected from each group just before glucose administration (0 min) and at 30, 60, and 120 min after glucose administration.

### 2.5. Plasma and Serum Biochemical Assays

Albino rats were sacrificed using isoflurane (5% W) anesthesia, and blood was collected by cardiac puncture. Serum was isolated by centrifugation (LABCENT 5000) at 3000 rpm 15 min as per standard protocols. Serum insulin, C-peptide, C-reactive protein (CRP), and Adenosine Deaminase (ADA) levels were assayed by the solid-phase enzyme-linked immunosorbent assay (ELISA) (Millipore; Billerica, USA, cat. no.: EZRMI-13K). Method described by Wilson and Spiger [39] was used to determine the lipid profile. Lipoproteins were fractionated by a dual precipitation technique of and high-density lipoprotein (HDL) and low-density lipoprotein (LDL) [40]. Total cholesterol (TC) and triglycerides (TGs) were measured using commercially available kits (Abcam, UK) following the manufacturer's protocol. Glycosylated hemoglobin (HbA1C) levels were estimated by the method described by Rao and Pattabiraman [41].

### 2.6. Pancreatic and Liver Homogenate Assays

Liver and pancreas homogenates, 10% (w/v), were prepared in a potassium phosphate buffer 0.05 M, pH 7.4. Elvehjem homogenizer (Dominique Dutscher, Issy-les-Moulineaux, France) was used. The homogenates were centrifuged for 10 min at 3000 × g at 4°C, and the supernatant aliquots were saved and stored at -20°C. The homogenate protein content was measured according to the method described by Lowry et al. [42]. Superoxide dismutase (SOD) activity was measured by rate of inhibition of pyrogallol autooxidation on a spectrophotometer at 470 nm according to the method of Marklund and Marklund [43]. SOD activity was expressed as units per milligram protein. The method described by Rotruck et al. was used to determine the glutathione peroxidase (GPx) level. It was calculated by the rate of NADPH oxidization spectrophotometrically at 340 nm [44]. GPx activity was presented as micrograms (*μ*g) of reduced glutathione (GSH) expended/min/mg protein. The method described by Sinha was used to quantify the catalase (CAT) activity as estimated by monitoring the decomposition of H_2_O_2_ spectrophotometrically at 240 nm [45]. Levels of tumor necrosis factor-alpha (TNF-*α*; cat. no.: CSB-E04741m), interleukin-1 beta (IL-1*β*; cat. no.: CSB-E04621m). and interleukin-6 (IL-6; cat. no.: CSB-E04627m) were assessed in the pancreas supernatant by ELISA using anti-mouse antibody (CUSABIO Life Sciences, Wuhan, China) based on the manufacturer's instructions.

Liver xanthine oxidase level was determined using methods described by Mohamed et al. [46]; membrane GLUT4 transporters were quantified using ELISA kit procured from Life Science Inc. (USA) following the manufacturer's instructions [47]; glucokinase (GCK) level was quantified using the ELISA method in accordance with the instructions of the GCK ELISA kit (provided by Beijing Lvyuan Bode Biotechnology Co., Ltd.)

### 2.7. Statistical Analysis

Results are presented as means ± SEM, one-way ANOVA, parametric (unpaired Student's *t*-test), or nonparametric (Mann-Whitney *U*-test) unpaired tests also using GraphPad Prism® version 6.00 for Windows (San Diego, CA, USA). The level of significance was set at *p* < 0.05.

## 3. Results

### 3.1. Body Weight and Pancreatic Organosomatic Index Evaluation

**Figure 1:**
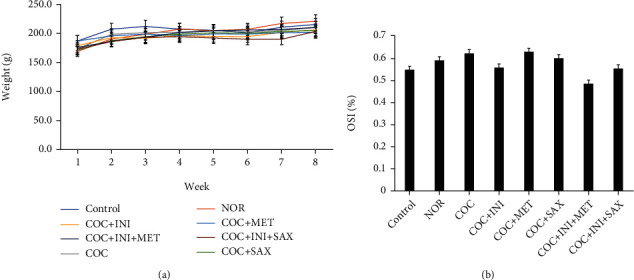
(a) The effect of treatment groups on weekly body weight monitoring. There was no statistically significant alteration in the weight of the treatment groups when compared with the control at *p* ≤ 0.05; mean ± SEM, *n* = 8. (b) The organosomatic index (OSI) carried out on the pancreas showed no statistical difference in the weight of the organ in the treatment group when compared with the control *p* ≤ 0.05; mean ± SEM, *n* = 8.

The 9-week study featured a weekly monitoring of the animal weight per group (*n* = 8). There was no statistically significant difference across the treatment group (Figure 1(a)). This trend was similar in the organosomatic index, which also showed no significant alteration in this index across the experimental groups (Figure 1(b)).

### 3.2. Fasting Blood Glucose Level and Oral Glucose Tolerance Test

**Figure 2:**
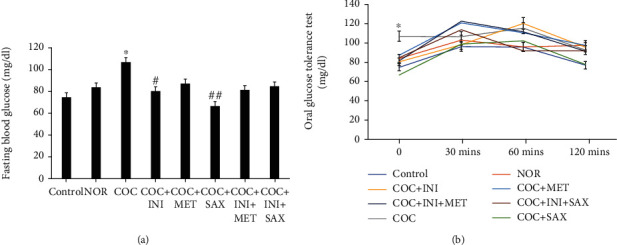
(a) COC caused a significant increase in the fasting blood glucose level when compared with control (^∗^*p* ≤ 0.05); this increase was significantly reversed by administration of SAX (^##^*p* ≤ 0.01). INI administration also reduced the FBG significantly (^#^*p* ≤ 0.05); mean ± SEM, *n* = 8. (b) The oral glucose tolerance test showed an initial increase in the blood glucose. However, there was no significant effect on the tolerance test among the tested group in the course of the 120 mins tolerance test; mean ± SEM, *n* = 6.

There was a significant increase in the fasting blood glucose level of the COC group (*p* ≤ 0.05); this alteration was however reversed in the INI group (*p* ≤ 0.05) and the SAX group (*p* ≤ 0.01) (Figure 2(a)). The OGTT showed no significant effect across the treatment groups except for the initial spike in glucose level observed at the 0 min timepoint (Figure 2(b)).

### 3.3. Serum Lipid Profile Analysis

**Figure 3:**
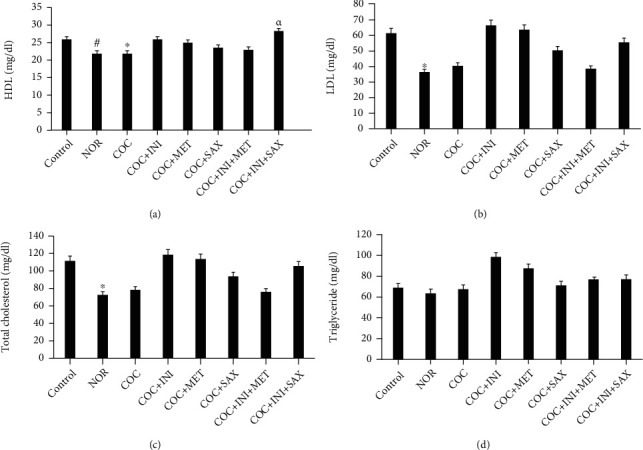
(a) COC caused a significant decrease in HDL level when compared with control (^∗^*p* ≤ 0.05); this was also observed in the NOR group (^#^*p* ≤ 0.05). INI+SAX significantly (*^α^p* ≤ 0.05) increased the HDL level when compared with the COC group. (b, c) A significant reduction in the LDL and total cholesterol levels of the NOR-treated group, respectively (^∗^*p* ≤ 0.05) (mean ± SEM, *n* = 8).

HDL levels were significantly reduced in both COC (*p* ≤ 0.05) and NOR (*p* ≤ 0.05) groups. This reduction was reversed in the INI+SAX group (*p* ≤ 0.05) (Figure 3(a)). The NOR group showed a significant reduction in both LDL and total cholesterol levels (*p* ≤ 0.05) (Figures 3(b) and 3(c), respectively). There was no significant alteration in the triglyceride level across the experimental groups.

### 3.4. Evaluation of Insulin, C-Peptide, C-Reactive Protein, and ADA Levels

**Figure 4:**
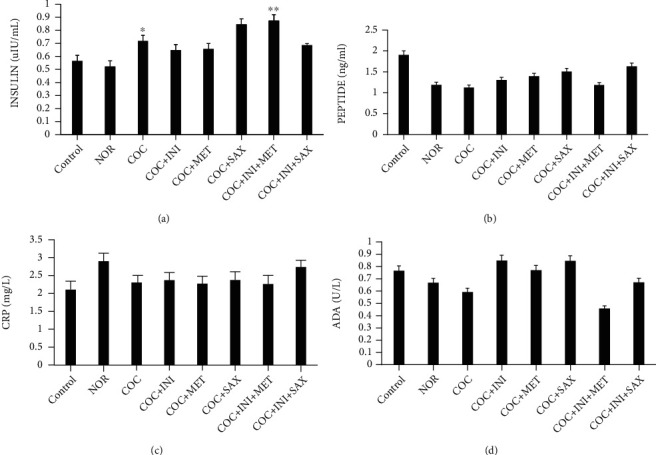
(a) COC caused a significant increase in insulin level when compared with control (^∗^*p* ≤ 0.05); this was also observed in the INI+MET group (^∗∗^*p* ≤ 0.01) when compared with the COC group. (b, c) No significant difference across the groups for C-peptide and CRP, respectively (mean ± SEM, *n* = 8).

Insulin level was significantly increased in the COC group (*p* ≤ 0.05) when compared with the control; this level was further increased in the INI+MET group (*p* ≤ 0.01) (Figure 4(a)). The C-peptide, C-reactive protein, and ADA levels were not significantly different across the experimental groups (Figures 4(b)–4(d), respectively).

### 3.5. Assessment of Antioxidant Parameters on Pancreas Homogenate

**Figure 5:**
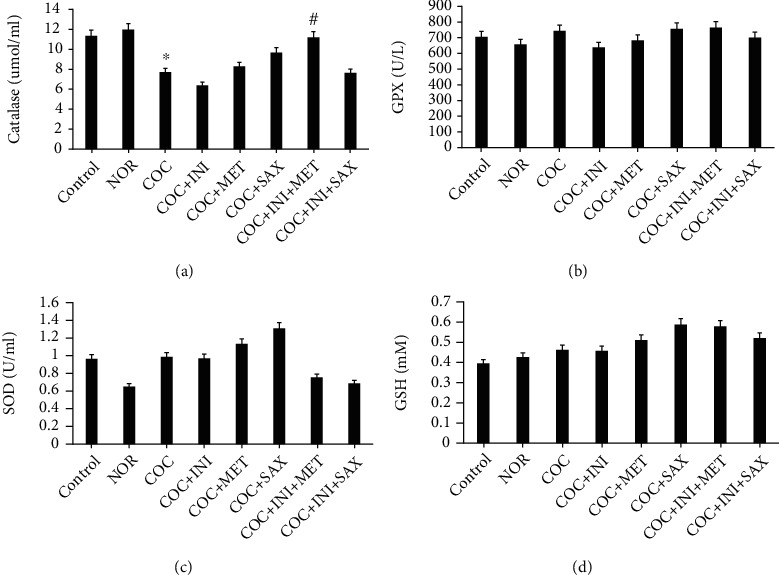
(a) COC caused a significant decrease in catalase level when compared with control (^∗^*p* ≤ 0.05); this decrease was reversed in the INI+MET group (^#^*p* ≤ 0.01) when compared with the COC group. (b, c) No significant difference across the groups for SOD and GPX, respectively (mean ± SEM, *n* = 6).

**Figure 6:**
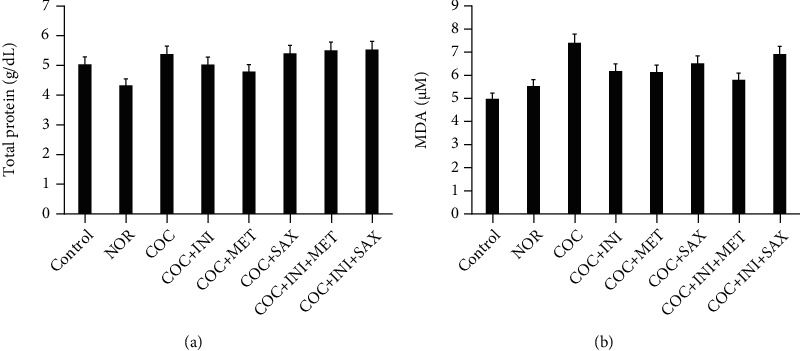
(a, b) No significant alterations across the test groups for total protein and MDA levels, respectively; *p* ≤ 0.05; mean ± SEM, *n* = 6.

Catalase level was significantly reduced in the COC group when compared with the control (*p* ≤ 0.05); this reduction was reversed in the INI+MET group (*p* ≤ 0.01). SOD, GPx, and GSH levels were not significantly affected across the experimental groups (Figures 5(b)–5(d)); total protein and MDA levels were not also significantly affected across the experimental groups (Figures 6(a) and 6(b)).

### 3.6. Assessment of TNF-*α*, IL-6, and IL-1*β* Levels in Pancreas Homogenate

**Figure 7:**
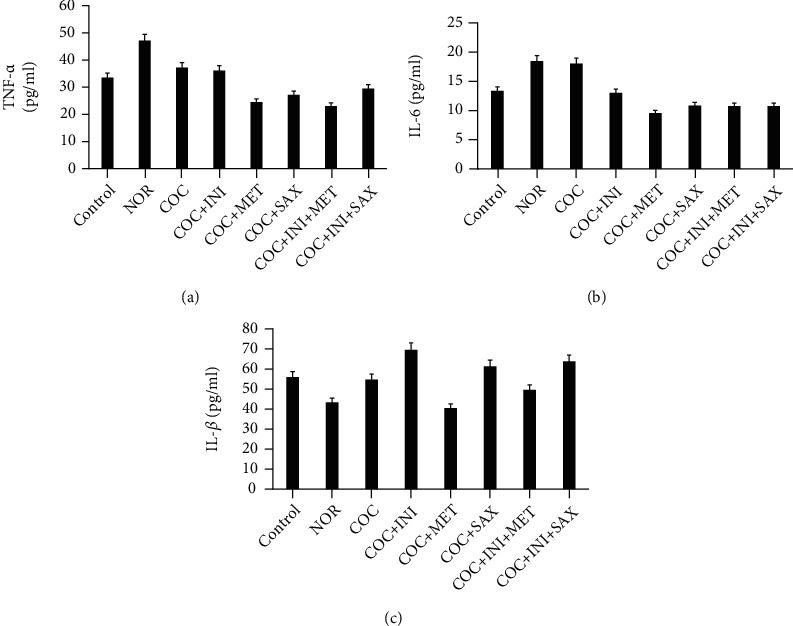
(a–c) No statistically significant alterations across the test groups for the inflammatory mediators: TNF-*α*, IL-6, and IL-1*β*, respectively. However, there is a nonstatistical increase in the level of TNF-*α* and IL-6 in the COC group and a subsequent reduction in the MET and INI+MET groups for both mediators, *p* ≤ 0.05; mean ± SEM, *n* = 6.

Figures 7(a)–7(c) show that levels of TNF-*α*, IL-6, and IL-1*β* were not significantly different statistically in comparison with the control. However, nonstatistical compares suggest an increase in the TNF-*α* levels in the COC group and a subsequent reduction in the MET and INI+MET groups.

### 3.7. Evaluation of Glucokinase, G6P, and GLUT4 Levels in Liver Homogenate

**Figure 8:**
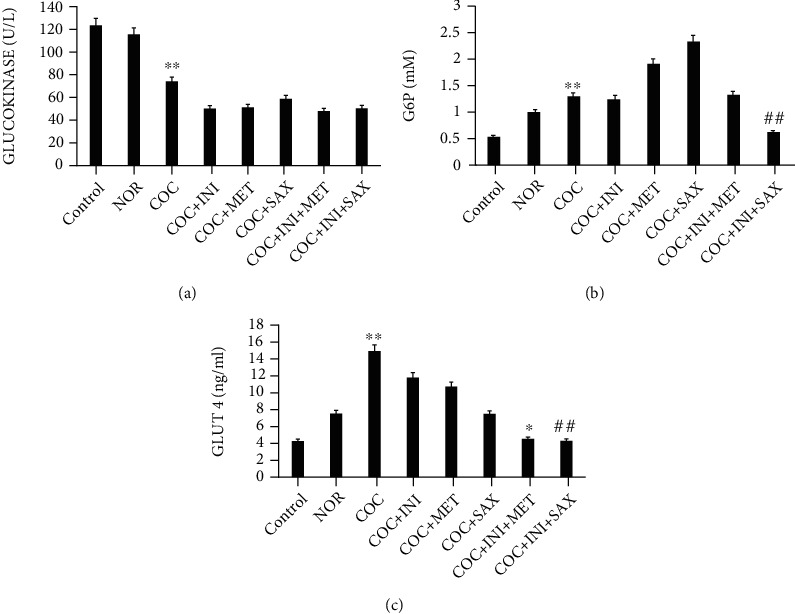
(a) COC caused a significant decrease in glucokinase level when compared with control (^∗∗^*p* ≤ 0.01); this decrease was not reversed by any of the treatment group (*p* ≤ 0.01) when compared with the COC group. (b) COC increased the G6P level (^∗∗^*p* ≤ 0.01); this effect was reversed significantly by the INI+SAX group (^##^*p* ≤ 0.01). (c) COC increased the GLUT4 levels significantly (^∗∗^*p* ≤ 0.01); this effect was reversed in INI+MET (^∗^*p* ≤ 0.05) and INI+SAX (^##^*p* ≤ 0.01) (mean ± SEM, *n* = 6).

COC caused a significant decrease in glucokinase level when compared with control (^∗∗^*p* ≤ 0.01); this decrease was not reversed by any of the treatment group when compared with the COC group (Figure 8(a)). COC increased the G6P level (^∗∗^*p* ≤ 0.01). This effect was reversed significantly by the INI+SAX group (^##^*p* ≤ 0.01) (Figure 8(b)). COC increased the GLUT4 levels significantly (^∗∗^*p* ≤ 0.01); this effect was reversed in INI+MET (^∗^*p* ≤ 0.05) and INI+SAX (^##^*p* ≤ 0.01) (Figure 8(c)).

### 3.8. Evaluation of Plasma HbA1c and Liver Xanthine Oxidase Levels

**Figure 9:**
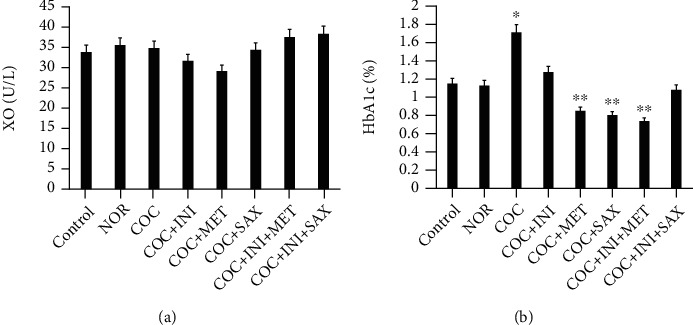
(a) No significant alterations across the test groups for xanthine oxidase (XO), *p* ≤ 0.05; (b) COC increased significantly the level of HBA1c when compared with the control (^∗^*p* ≤ 0.05); this effect was reversed significantly in the MET, SAX, and INI+MET groups (^∗∗^*p* ≤ 0.01); mean ± SEM, *n* = 6; *n* = 8.

Glycated hemoglobin (HbA1c) levels assayed for in the plasma showed a significant increase in the COC group (^∗^*p* ≤ 0.05); this effect was reversed significantly in the MET, SAX, and INI+MET groups (^∗∗^*p* ≤ 0.01) (Figure 9(a)). Xanthine oxidase levels were not significantly different across the experimental groups (Figure 9(b)).

## 4. Discussion

A constellation of metabolic abnormalities which features hyperglycemia, hyperinsulinemia, and hyperlipidemia among other disorders was initially termed as “Insulin resistance syndrome” [48]. These metabolic abnormalities, generally now referred to as metabolic syndrome (MetS), are increasing at an alarming rate, becoming a major public and clinical problem worldwide [49]. MetS has been linked to cardiovascular dysfunction and a major risk factor in the development of type 2 diabetes mellitus [50, 51]. This present study sets out to unravel the effect of combined oral contraceptives (COCs) on specific elements of MetS and the possible benefits of intranasal insulin (INI), metformin (MET), and saxagliptin (SAX) in various combination regimens in the management of this syndrome. It is expected that the results from this study shed light on the underlying therapeutic intervention for this disorder occasioned by a chronic administration of COC in adult female albino Wistar rats.

Body weight changes and particularly body mass index (BMI) have been linked directly to metabolic syndrome status in humans [52], with a particular interest in the relationship between MetS and waist circumference [53, 54]. Weekly monitoring of the experimental groups showed that the chronic exposure to combined oral contraceptives did not affect the animal body weight during the study. In their study, Ghezzi et al. postulated that weight loss or distortion takes place in the early developmental stages of the animals, and this study attributed the unlikelihood of weight gain at maturity to a physiological decline in the development of the animals at adulthood [55]. This phenomenon is in tandem with our findings, since adult female Wistar rats were used. Organosomatic index of the pancreas was also in line with the body weight changes, and this is expected due to the fact that insulin resistance may not be a sign of toxicity to the pancreas, since this is not insulin insufficiency as seen in the etiology of type 1 diabetes mellitus [56]. Organosomatic index is a more definitive examination of organ weight which shows the proportional sizes of organs and depicts the status of organ systems. Organ sizes relative to body weight may change faster than organism weight and are an important indicator of organ toxicity. Organosomatic indices have been globally accepted as a pointer of organ-system integrity of animals [57].

High blood glucose (hyperglycemia) and hyperinsulinemia are major features of metabolic syndrome and particular risk factors in type 2 diabetes mellitus [50]. Use of combined oral contraceptive induces significant increases in both fasting blood glucose and insulin levels; this establishes the fact that the continuous use of this contraceptive predisposes to type 2 diabetes mellitus and cardiovascular dysfunctions. Treatment with saxagliptin markedly reduced the fasting blood glucose levels, and a reduction in glycemic level was also observed in the intranasal insulin group. Insulin resistance depicted by hyperinsulinemia can dysregulate glucose metabolism generating chronic hyperglycemia, leading to the onset of cardiac related abnormalities [58]. A proper management of the elements of MetS in women of reproductive age can prevent the development of cardiovascular diseases, which is the global leading cause of death [59]. C-reactive peptide, C-peptide, and ADA levels were not significantly affected; also, the oral glucose tolerance test was not affected by the combined oral contraceptives.

Lipid profile analysis, with particular focus on the HDL, LDL, and triglyceride level, has been specifically linked to metabolic syndrome [60]. Combined oral contraceptive use reduced the level of HDL; this reduction was reversed by intranasal insulin and saxagliptin combined therapy; LDL and total cholesterol levels were reduced by the administration of norethindrone; however, triglyceride levels were not affected by the combined contraceptives. Insulin resistance alters systemic lipid metabolism leading to the development of dyslipidemia [61]; the 3 popular lipid-related aberrations are as follows: (1) upregulated levels of plasma triglycerides, (2) low levels of high-density lipoprotein, and (3) the presence of low-density lipoproteins. These features, in combination with dysfunction of the endothelial, which can also be caused by disrupted insulin signaling, are important underlying factors to formation of atherosclerotic plaques [58].

The association between oxidative stress and insulin resistance has been reported to be a predisposing link to cardiovascular diseases [62]. Pancreatic homogenate antioxidant assay showed that the level of catalase enzyme was significantly reduced consequent upon exposure to combined oral contraceptives; this reduction was reversed by treatment with intranasal insulin and metformin combination therapy. Catalase is an antioxidant enzyme which catalyzes the breakdown of hydrogen peroxide to water and oxygen. It is crucial for the protection of cells from oxidative damage by reactive oxygen species (ROS) [63]. Deficiency or malfunction of catalase is postulated to be related to the pathogenesis of many age-associated degenerative diseases like diabetes mellitus, hypertension, anemia, and vitiligo [64]. Other antioxidant parameters such as SOD, GSH, GPx, and MDA levels were not affected significantly by treatment with the combined oral contraceptive in this study.

Glycated hemoglobin (HbA1c) levels have been used as a diagnostic parameter for profiling insulin resistance in patients and a probe for type 2 diabetes mellitus [65]. We checked for the effect of combined oral contraceptive treatment on HbA1c and discovered a significantly high level (*p* ≤ 0.05); this effect was reversed in the MET, SAX, and INI+SAX groups (*p* ≤ 0.01), suggesting their efficacy in the management of MetS caused by the use of combined oral contraceptive which could subsequently lead to the prevention of type 2 DM and cardiotoxicity. The formation of the sugar-hemoglobin linkage indicates the presence of excessive sugar in the bloodstream, often indicative of diabetes [66]. HbA1C is of particular interest because it is easy to detect. Liver GLUT4 level was upregulated in the COC group and subsequently reversed in the INI+MET and INI+SAX groups. However, we suggest this could be as a result of the length of the study; we expected a reduction in the level of GLUT4 activity. During the normal physiology of glucose-stimulated secretion of insulin, beta-cells release insulin into circulation, activating GLUT4-dependent glucose uptake into peripheral tissue. Glucokinase level was significantly reduced in the COC group; glucose-6-phosphate levels were increased in the same group but subsequently reduced in the INI+SAX group. Glucokinase converts glucose to glucose-6-phosphate which may be converted to glycogen for storage [67]. Inflammatory cytokines such as TNF-*α*, IL-6, and IL-1*β* play significant roles in the etiology of metabolic syndrome. Scientific evidence suggests that obesity, in particular chronic visceral adiposity, is associated with inflammatory markers including interleukin-6 (IL-6) and tumor necrosis factor-alpha (TNF-*α*) [68]. Studies have shown that IL-6 and TNF-*α* are secreted from infiltrated macrophages into adipose tissue and have critical roles in MetS, insulin resistance, nonalcoholic fatty liver disease, and atherogenesis [69]. Our study showed that these cytokines (TNF-*α* and IL-6) were affected without statistical significance. Nonstatistically significant increase in TNF-*α* and in COC-treated group was reversed by administration of MET and combined INI+MET. These suggest an early stage or development of MetS can be arrested with these therapeutic regimens.

## 5. Conclusion

Findings from this study show that chronic combined oral contraceptive treatment predisposes female albino rats to metabolic syndrome which features hyperglycemia, dyslipidemia, hyperinsulinemia, etc., all of which were featured in this present study. The use combination therapies particularly involving saxagliptin and metformin corrected some of these metabolic abnormalities that have a direct link to glucose metabolism. Thus, the metabolic syndrome status of combined oral contraceptive users should be closely monitored, and therapeutic interventions should be employed to prevent development of type 2 DM and other cardiovascular pathologies. A clinical study should be engaged in order to establish the translational relevance of these findings in women of reproductive age.

## Data Availability

The datasets used and/or analyzed during the current study are available from the corresponding author upon request.
